# Supplementary material for: An open-source framework for large-scale, flexible evaluation of biomedical text mining systems
Source: J Biomed Discov Collab. 2008 Jan 29;3:1. doi: 10.1186/1747-5333-3-1 (PMC2276192; doi:10.1186/1747-5333-3-1)
Supplement: Additional file 1 — Evaluation platform source code. The additional file contains the software evaluation framework discussed in this paper. The most current release can be downloaded from SourceForge.net [1]. See the accompanying README file for instructions on installation and use. Also included in the distribution is a collection of UIMA wrappers for some commonly used BioNLP tools and annotated corpora. [file 1747-5333-3-1-S1.zip › bionlp_uima_repository-1.0.1/docs/api/src-html/edu/uchsc/ccp/util/external/opennlp/OpenNLPSentenceSegmenter.html]

```
001    package edu.uchsc.ccp.util.external.opennlp;
002    
003    import java.io.IOException;
004    import java.util.ArrayList;
005    import java.util.List;
006    
007    import opennlp.tools.lang.english.SentenceDetector;
008    import edu.uchsc.ccp.util.nlp.annotation.AnnotationSet;
009    import edu.uchsc.ccp.util.nlp.annotation.Annotator;
010    import edu.uchsc.ccp.util.nlp.annotation.TextAnnotation;
011    import edu.uchsc.ccp.util.nlp.mention.ClassMention;
012    import edu.uchsc.ccp.util.nlp.mention.ClassMentionTypes;
013    
014    public class OpenNLPSentenceSegmenter {
015    
016        private SentenceDetector sd;
017    
018        private final Annotator annotator = new Annotator(55,"OpenNLP","OpenNLP","OpenNLP");
019        private final AnnotationSet annotationSet = new AnnotationSet(-1,"Default Set","Default Set");
020        
021        public OpenNLPSentenceSegmenter(String modelFile) throws IOException {
022            this.sd = new SentenceDetector(modelFile);
023        }
024    
025        public List<TextAnnotation> segmentIntoSentences(String inputText, String documentID) {
026            List<TextAnnotation> sentenceAnnotations = new ArrayList<TextAnnotation>();
027            String[] sentences = sd.sentDetect(inputText);
028    
029            int numSentences = sentences.length;
030            
031            int startIndex = 0;
032            int[] positions = sd.sentPosDetect(inputText);
033            for (int pos : positions) {
034                int endIndex = pos;
035                String sentence = inputText.substring(startIndex,endIndex);
036                
037                while (sentence.endsWith(" ")) {
038                    endIndex--;
039                    sentence = inputText.substring(startIndex,endIndex);
040                }
041                
042                TextAnnotation sentenceAnnot = createSentenceAnnotation(startIndex, endIndex, sentence, documentID);
043                sentenceAnnotations.add(sentenceAnnot);
044                
045                startIndex = pos;
046            }
047    
048            if (startIndex < inputText.length()) {
049            String finalSentence = inputText.substring(startIndex,inputText.length());
050            TextAnnotation sentenceAnnot = createSentenceAnnotation(startIndex, inputText.length(), finalSentence, documentID);
051            sentenceAnnotations.add(sentenceAnnot);
052            }
053            
054            /* safety check for correct number of sentences */
055            if (sentenceAnnotations.size() != numSentences) {
056                error("Unexpected number of sentences returned. Expected: " + numSentences + " but got: " + sentenceAnnotations.size());
057            }
058            
059            return sentenceAnnotations;
060        }
061        
062        
063        private TextAnnotation createSentenceAnnotation(int spanStart, int spanEnd, String coveredText, String documentID) {
064            ClassMention cm = new ClassMention(ClassMentionTypes.SENTENCE);
065            TextAnnotation ta = new TextAnnotation(spanStart, spanEnd, coveredText,annotator, annotationSet, -1, -1, documentID, -1, cm );
066            return ta;
067        }
068        
069        private void error (String message) {
070            System.err.println("ERROR -- OpenNLPSentenceSegmenter: " + message);
071        }
072        
073        /**
074         *
075         * @param args
076         * args[0] = sentence detection model file - trained on wsj
077         */
078        public static void main(String[] args) {
079            String englishModelFile = args[0];
080            
081            try {
082                OpenNLPSentenceSegmenter osd = new OpenNLPSentenceSegmenter(englishModelFile);
083                
084                String abstractText = "Angiogenesis has long been established as a key element in the pathophysiology of tumor growth and metastasis. Increasingly, new molecularly targeted antiangiogenic drugs are being developed in the fight against cancer. These drugs bring with them a need for an accurate means of diagnosing tumor angiogenesis and monitoring response to treatment. Imaging techniques can offer this in a noninvasive way, while also providing functional information about the tumor. Among the many clinical imaging techniques available, MRI alone can provide relatively good spatial resolution and specificity, without ionizing radiation and with limited side effects. Arterial spin labeling (ASL) and blood oxygenation level-dependent (BOLD) imaging techniques can be employed to confer indirect measures of angiogenesis, such as blood flow and blood volume, without the need for external contrast agents. Dynamic contrast-enhanced (DCE)-MRI is rapidly emerging as a standard method for directly measuring angiogenesis during angiogenesis-inhibitor drug trials. As macromolecular MR contrast agents become available, they will inevitably be utilized in the assessment of tumor perfusion and vessel permeability. Meanwhile, technological advances have made imaging at a molecular level a possibility. They have brought the potential to directly target MR contrast agents to markers of angiogenesis, such as the alpha(v)beta(3) integrin. Before this is used clinically, however, substantial gains in sensitivity brought about by improved coils, pulse sequences, and contrast agents will be needed. Herein we discuss the techniques currently available for MRI of angiogenesis, along with their respective advantages and disadvantages, and what the future holds for this evolving field of imaging. J. Magn. Reson. Imaging 2007. (c) 2007 Wiley-Liss, Inc.";
085                
086                List<TextAnnotation> sentenceAnnots = osd.segmentIntoSentences(abstractText, "12345");
087                
088                for (TextAnnotation ta : sentenceAnnots) {
089                    ta.printAnnotation(System.out);
090                }
091                
092            } catch (IOException e) {
093                e.printStackTrace();
094            }
095        }
096    
097    }
```
